# Isolation and characterisation of human pulmonary microvascular endothelial cells from patients with severe emphysema

**DOI:** 10.1186/1465-9921-14-23

**Published:** 2013-02-20

**Authors:** Laura S Mackay, Sara Dodd, Iain G Dougall, Wendy Tomlinson, James Lordan, Andrew J Fisher, Paul A Corris

**Affiliations:** 1 Institute of Cellular Medicine, Newcastle University, Medical School, Framlington Place, Newcastle upon Tyne NE2 4HH, UK; 2 Bioscience Department, Astra Zeneca R&D Charnwood, Bakewell Rd, Loughborough LE11 5RH, UK

**Keywords:** Primary cell isolation, Microvasculature, Emphysema

## Abstract

**Background:**

Loss of the pulmonary microvasculature in the pathogenesis of emphysema has been put forward as a credible alternative to the classical inflammatory cell driven proteolysis hypothesis. Mechanistic studies in this area have to date employed animal models, immortalised cell lines, primary endothelial cells isolated from large pulmonary arteries and non-pulmonary tissues and normal human pulmonary microvascular endothelial cells. Although these studies have increased our understanding of endothelial cell function, their relevance to mechanisms in emphysema is questionable. Here we report a successful technique to isolate and characterise primary cultures of pulmonary microvascular endothelial cells from individuals with severe emphysema.

**Methods:**

A lobe of emphysematous lung tissue removed at the time of lung transplantation surgery was obtained from 14 patients with severe end-stage disease. The pleura, large airways and large blood vessels were excised and contaminating macrophages and neutrophils flushed from the peripheral lung tissue before digestion with collagenase. Endothelial cells were purified from the cell mixture via selection with CD31 and UEA-1 magnetic beads and characterised by confocal microscopy and flow cytometry.

**Results:**

Successful isolation was achieved from 10 (71%) of 14 emphysematous lungs. Endothelial cells exhibited a classical cobblestone morphology with high expression of endothelial cell markers (CD31) and low expression of mesenchymal markers (CD90, αSMA and fibronectin). E-selectin (CD62E) was inducible in a proportion of the endothelial cells following stimulation with TNFα, confirming that these cells were of microvascular origin.

**Conclusions:**

Emphysematous lungs removed at the time of transplantation can yield large numbers of pulmonary microvasculature endothelial cells of high purity. These cells provide a valuable research tool to investigate cellular mechanisms in the pulmonary microvasculature relevant to the pathogenesis of emphysema.

## Background

Lung endothelial cell injury is hypothesised to be a key event in the pathogenesis of emphysema [1,2] and forms an increasingly credible “microvascular hypothesis” as an alternative to the classical hypothesis in which inflammatory cells are seen as the orchestrators of tissue destruction [3]. A number of models have been employed to investigate the role of the pulmonary endothelium ranging from *in*-*vitro* cellular systems to *in*-*vivo* animal models. Early cellular studies were based on large vessel endothelial cells, typically from the main pulmonary trunk, or used human umbilical vein endothelial cells (HUVECs) as a surrogate for the lung microvasculature [4]. Immortalised human cells lines have also been used as they provide a stable cell population and are easily expanded for use in a range of assays. However such cells, which evade the normal controls within the cell cycle [5], do not always express markers characteristic of the tissue in which they originated [6,7] and their responses *ex vivo* may not reflect the true *in vivo* response of cells to injury, thus limiting their relevance [8,9]. Pulmonary microvascular endothelial cells, which form the luminal barrier of intra-acinar arterioles and venules and the alveolar capillary network have also been isolated from bovine [10], ovine [11] and rodent lungs [12] which provide more biologically relevant models in which endothelial cell responses to injury can be studied. Although these systems may not accurately reflect human cellular responses, they have facilitated the development of methods to effectively isolate lung microvascular endothelial cells (LMVECs) from normal human tissue [13-15] and such cells are now available from a number of commercial suppliers. These commercially available primary LMVECs have the advantage of being fully compliant with regulatory legislation and information regarding patient age and in some cases smoking status is available. However, it is impossible to determine whether the individuals from whom cells were isolated had normal pulmonary function or whether they had any pre-existing lung disease. The ability to compare cellular responses in disease free individuals with those who have developed severe disease is very attractive given the observation that only about 20% of individuals who smoke develop emphysema [16] suggesting that the pathology reflects an individual’s disordered cellular response to the injury rather than the injury *per se*. Thus, comparing how lung microvascular endothelial cells from susceptible individuals behave in contrast to lung microvascular endothelial cells isolated from individuals free from emphysema may provide unique insights into the cellular responses to cigarette smoke which lead to emphysema.

In this study we present, to our knowledge, the first report of successful isolation of LMVECs from well characterised patients with severe emphysema. The cells from emphysematous lung were then characterised and compared with LMVECs from pathologically normal lung tissue derived from patients undergoing isolated lung cancer resection surgery and with commercially available LMVECs.

### Subjects

Ethical approval to obtain emphysematous tissue from patients undergoing lung transplantation was granted by the Northumberland Local Research Ethics Committee (REC reference 06/Q0902/57). All patients awaiting lung transplantation for emphysema at Freeman Hospital, Newcastle Upon Tyne, UK were invited to take part in the study. Patients gave informed consent to donate their explanted lung for research purposes. The study was performed in accordance with ICH-GCP.

Ethical approval to obtain normal tissue from patients undergoing lobectomy/ pneumonectomy was granted by County Durham and Tees Valley 2 Research Ethics Committee (REC reference 09/H0908/35). Patients between the ages of 18–75 years undergoing lobectomy/ pneumonectomy with no evidence of emphysema/ fibrosis on radiology or pulmonary function testing were invited to take part in the study. The study was performed in accordance with ICH-GCP.

Clinical data including age, body mass index and pulmonary function tests were obtained from each individual who donated tissue. Smoking status and smoking history from each patient was also obtained. Those with emphysema were also categorized according to the updated GOLD criteria (2003) [17,18].

## Methods

### Obtaining diseased lung tissue

At the time of transplantation, the lung was inspected to confirm the macroscopic pathology was in keeping with pre-operative diagnosis and to exclude any unexpected pathology. A lobe or part of a lobe was then dissected and stored at 4°C until clinical pathology assessment (which was typically performed within 12 hours). Following routine clinical pathology, blocks of tissue were fixed in neutral buffered formalin for investigative pathology. The remaining tissue (typically around 50 g) was used immediately for cell isolation.

### Obtaining normal tissue

The operating surgeon performing lobectomy for suspected lung cancer identified a wedge of normal tissue within the tissue removed at surgery but discrete from the tumor resection margins. This was dissected from the remaining tissue and tumour and placed in media. Both samples were transported to clinical pathology where the wedge sample was inspected and once confirmed to be free from disease, was used immediately for cell isolation. Tissue samples ranged from 5-30 g.

### Cell isolation

Contaminating macrophages were removed via repeated inflation of the tissue with sterile phosphate buffered saline (PBS). The pleura, visible arterioles, bronchioles and venules were then dissected to prevent overgrowth with mesothelial and epithelial cells and reduce contamination with macrovascular endothelial cells. The remaining peripheral lung tissue was washed in RPMI containing 10% fetal calf serum (FCS) and 1% penicillin streptomycin and amphotericin (PSA) and finely chopped (1-2 mm^2^ pieces). The tissue pieces were then washed on a 40 μm filter to remove red blood cells before incubation with 0.2% type II collagenase (CLS-2, Worthington) in RPMI containing 0.1% bovine serum albumin (BSA) for 2 hours on a roller at room temperature. Following incubation, the suspension was filtered on a 400-500 μm mesh and then a 100 μm sterile filter. The filtrate was centrifuged (250 *g* for 5 minutes). The supernatant was discarded and resulting cell pellet re-suspended in endothelial growth MV2 media (Promocell) containing 1% PSA. An automated cell count was performed and cells plated onto flasks pre-coated with 0.2% gelatin (w/v in MilliQ water, coated for 30 min at room temperature, excess gelatin solution was removed before cell addition) at approximately 10,000 cells/cm^2^. Cells were cultured at 37°C in the presence of 5% CO2. Non-adherent cells were removed after 24 hours in culture by gentle flushing with PBS over the flasks. MV2 media was replaced every 3–4 days.

### Endothelial cell purification

When the cells reached approximately 80% confluence, they were passaged using cell dissociation solution (Sigma) and separated from any contaminating fibroblast and epithelial cells using CD31 Dynal beads (Invitrogen) and pre-prepared Ulex europaeus agglutinin-1 (UEA-1) coated Dynal beads. UEA-1 binds to the α-L-Fucosyl residues of glycoprotein present on the surface of human microvascular endothelial cells, thus in conjugation with magnetic beads allows the selection of endothelial cells from a mixed cell suspension [19]. The cells were re-suspended in PBS containing 0.1% BSA and 2 mM EDTA (Dynal Buffer) and 25 ul each of CD31 Dynal beads and UEA-1 coated beads were added. The cells/beads mixture was incubated on a rocker at 4°C for 20 minutes, to minimise non-specific binding. The beads were then washed in Dynal buffer and placed in a Dynal magnet. The bead negative fluid was discarded. After repeated washing and magnetic separation, the bead positive cells were counted and plated on 0.2% gelatin coated tissue culture flasks at approximately 3,000 cells/cm^2^ and incubated at 37°C in the presence of 5% CO_2_. Bead separation was performed over 3–5 passages of the cells until pure cobblestone cultures were obtained.

### Cryopreservation of cells

When cultures appeared free from contaminating cells, cells were cryopreserved in MV2 media (Promocell) containing 1% DMSO (Sigma). All emphysema cultures were cryopreserved and then later reanimated for characterisation and explorative experiments.

### Commercial human pulmonary microvascular endothelial cells

Commercial LMVECs were purchased from Promocell (C12281) and cultured after reanimation at 37°C with 5% CO_2_ using endothelial growth MV2 media (Promocell) supplemented with 1% PSA (as used with cells isolated from patients).

### Mycoplasma testing

All isolated cells and commercial cells were routinely tested for mycoplasma infection using Myco Alert kits (LT07-218, Lonza). Testing was carried out on all isolated cells prior to experimentation and on commercial cells on a monthly basis. The cells showed no evidence of mycoplasma infection.

### Phase contrast microscopy

Cells were grown to confluence and images taken on Canon image shot.

### Confocal microscopy

Cells were cultured on 18 mm glass coverslips in 12 well plates. At confluence, cells were washed in PBS and fixed in freshly prepared paraformaldehyde (4%). Following fixation, cells were quenched in 100 mM glycine for 30 minutes, before permeabilisation in PBS Triton X-100 (1% v/v) for 20 minutes. Following permeabilisation, cells were washed with PBS containing 0.2% tween (0.2% PBST) and PBS. After blocking with 5% BSA for 60 minutes, coverslips were incubated with primary antibodies (CD31 (Sc53411, Santa Cruz) Fibronectin (F3648, Sigma), αSMA, (F3777, Sigma) in 0.5% BSA overnight at 4°C. Cells were then washed as before with 0.2% PBST and PBS. Fluorochrome pre-conjugated secondary antibodies (FITC: Mouse (F2012) and TRITC: Rabbit (T6778), Sigma) were then applied (0.5% BSA) for 60 minutes and then washed in 0.2% PBST and PBS. The cells were then mounted with DAPI mounting medium (H-1200, Vector Labs) and viewed on a Leica Sp2UV laser scanning confocal microscope and analysed with software from Leica (LCS 2.61).

### Flow cytometry

Initial experiments to determine optimal concentrations of antibodies were conducted using microvascular endothelial cells purchased from Promocell. Each cell population was stained using the same conditions.

### CD31/CD90 characterisation

Cells at 70-80% confluence were used in all characterisation experiments. Cells were harvested using cell dissociation solution (Sigma) with approximately 100,000 cells per 100 ul used for each stain. Cells were washed and re-suspended in 100 ul PBS and incubated with FITC conjugated CD31 (#555445 BD Bioscience) and PE cy5 conjugated CD90 (# 555597 BD Bioscience) for 30 minutes at 4°C, to reduce non-specific binding. Cells were then washed in PBS, centrifuged at 250 *g* for 4 minutes, re-suspended in 200 ul PBS and analysed on FACS Scan (Becton Dickinson).

### CD62E characterisation

Cells were grown in 6 well plates and at 70-80% confluence were treated with TNFα (1 ng/ml). Following treatment cells were harvested using cell dissociation solution with approximately 100,000 cells per 100 ul used for each stain. Cells were washed and re-suspended in 100 ul PBS and incubated with APC conjugated CD62E (E-selectin) (#551144 BD Bioscience) for 30 minutes at 4°C, to reduce non-specific binding. Cells were then washed in PBS, centrifuged at 250 g for 4 minutes, re-suspended in 200 ul PBS and analysed on FACS Scan.

## Results

Cell isolation was attempted from lung tissue obtained from 16 patients (11 emphysema, 3 α1 anti-trypsin related emphysema and 2 normals) and was successful in 10 (71%) of the emphysema donors. Table 1 shows the baseline characteristics and clinical data from the 16 individuals in whom cell isolation was attempted. In addition to diagnosis, smoking history, body mass index (BMI) and spirometry measures were included. Those patients with emphysema were categorised according to disease severity based upon the GOLD classification. Cell yield from successful cultures is documented.

**Table 1:** Patient characteristics and cell yield

**Patient No:**	**Gender**	**Diagnosis**	**Age**	**BMI**	**Smoking History (Pack yrs)**	**FEV1 (%)**	**TLC (%)**	**KCO (%)**	**GOLD stage**	**Cell yield (passage number at cryopreservation)**
**1**	M	A1AT emphysema	46	28.7	15	18	133	15	IV	1.9 ×10^6^ cells (passage 4)
**2**	F	Emphysema	54	21.5	30	10	178	-	IV	2.5 ×10^6^ cells (passage 4)
**3**	F	Emphysema	53	20.9	30	22	131	32	IV	9.4 ×10^6^ cells (passage 5)
										5.4 ×10^6^ cells (passage 4)
**4**	F	Emphysema	51	20.8	30	28	150	33	IV	5.6 ×10^6^ cells (passage 4)
										28.8 ×10^6^ cells (passage 5)
**5**	F	Emphysema	46	20.2	20	21	150	43	IV	Unsuccessful
**6**	M	Emphysema	58	22.7	35	15	130	46	IV	Unsuccessful
**7**	F	Emphysema	59	21.8	25	34	155	38	III	12 ×10^6^ cells (passage 4)
**8**	M	Emphysema	44	23	15	14	138	69	IV	3.2 ×10^6^ cells (passage 4)
										21.5 ×10^6^ cells (passage 6)
**9**	F	Emphysema	60	28.3	20	26	95	25	IV	5.2 ×10^6^ cells (passage 6)
**10**	M	Emphysema	45	21.3	27	26	156	42	IV	5.4 ×10^6^ cells (passage 4)
**11**	M	Emphysema	55	20.8	55	17	130	24	IV	Unsuccessful
**12**	F	A1AT emphysema	40	26.1	25	16	136	33	IV	18.2 ×10^6^ cells (passage 6)
**13**	M	Emphysema	47	22.3	30	17	150	42	IV	13.9 ×10^6^ cells (passage 4)
**14**	M	A1AT emphysema	52	21.2	15	22	127	71	IV	Unsuccessful
**15**	F	Normal	68	-	15	104	-	-	N/A	1 ×10^6^ cells (passage 3)
**16**	F	Normal	65	-	40	80	-	-	N/A	Unsuccessful

### Phase contrast microscopy of isolated cells

**Figure 1:**
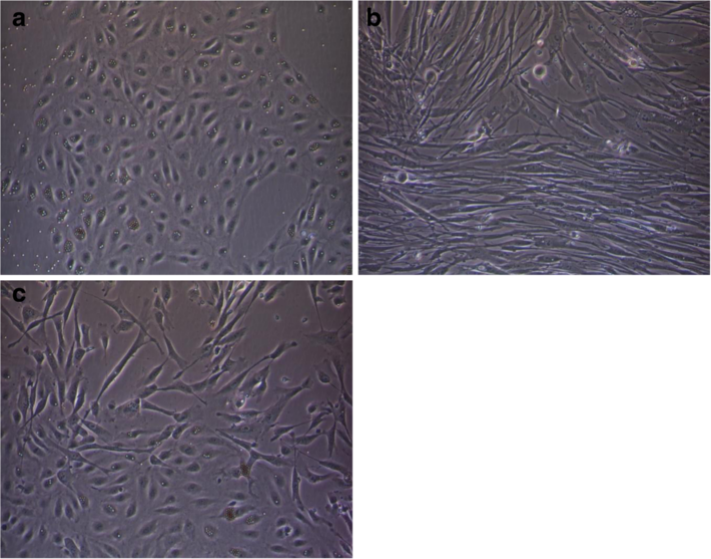
**Phase contrast microscopy of cells following the initial bead separation (Patient 5). a**) Cobblestone cells growing in colonies. **b**) Bead negative fraction showing elongated spindle cells. **c**) Areas showing a mixture of cobblestone cells and more elongated cells.

Prior to the first passage, cells in culture were a mixed population of elongated cells and cobblestone cells. Following the initial bead separation, at the first passage, the bead positive cells displayed cobblestone morphology and grew in a monolayer in colonies (Figure 1a). Beads could also be seen attached to many of the cobblestone cells. In contrast, the bead negative cells (Figure 1b) were elongated and spindle shaped and grew in sheets, becoming confluent more quickly. At the early passages a mixed population of cells was still present within areas of the bead positive fraction (Figure 1c) with some elongated cells growing together with cobblestone cells. For this reason, repeated bead separation was performed until cultures contained only cobblestone cells.

### Characterisation of cells via confocal microscopy

**Figure 2:**
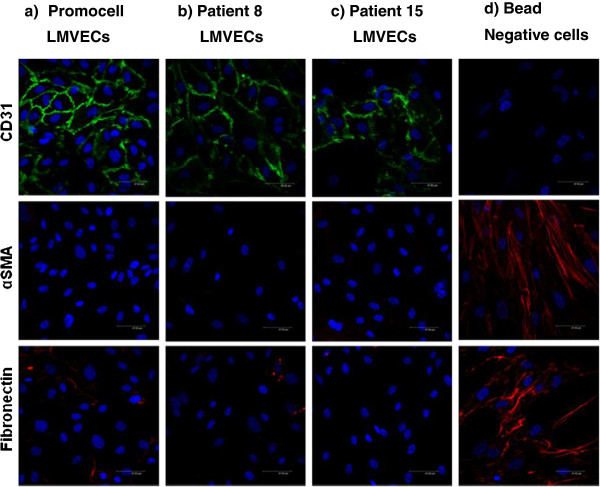
**Detection of immunocytochemical markers via confocal microscopy (CD31: FITC green, aSMA: TRITC red, DAPI: blue).** LMVECs (Promocell) (**a**) were compared to LMVECs isolated from patient 8 with emphysema (**b**) and LMVECs isolated from excess normal tissue (patient 15) (**c**). Cellular expression on these cells was compared with that on the bead negative fraction from excess normal tissue (**d**). All images were taken at X 63 magnification.

Cells stained positively for the endothelial cell surface marker CD31 (FITC green) (Figure 2a-c). Cells displayed contact inhibition with the formation of a lattice of tight junctions. The bead negative cells showed no CD31 staining (Figure 2d). The mesenchymal marker alpha smooth muscle actin (αSMA) (TRITC red) was absent on CD31 positive cells (Figure 2a-c) but was present on the CD31 negative fraction (Figure 2d) in an elongated spindle shaped pattern (red). CD31 positive cells (Figure 2a-c) also had very low levels of the intracellular matrix protein fibronectin, in contrast to CD31 negative cells (Figure 2d) which demonstrated high staining (red) in sheet like form.

### Characterisation of cells via flow cytometry

**Figure 3:**
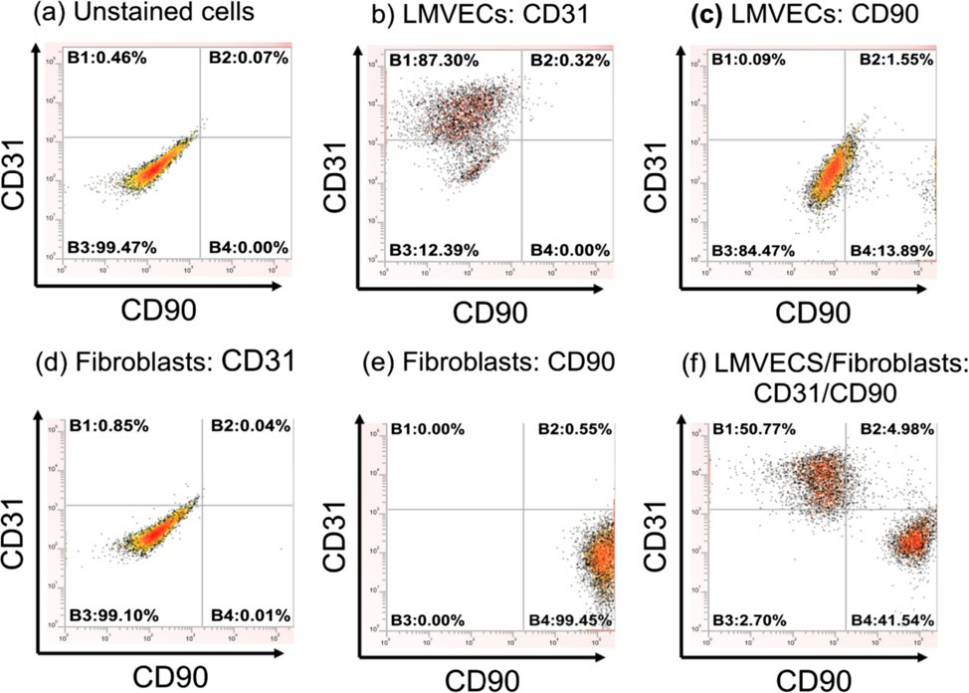
**Representative flow cytometry scatter plots showing CD31 and CD90 staining.** Unstained mixed cell population, LMVECs (Promocell) and fibroblasts (**a**). Endothelial cells stain positively for CD31 (**b**) and negatively for CD90 (**c**). Fibroblasts stain negatively for CD31 (**d**) but strongly positive for CD90 (**e**). Mixed Endothelial cells and fibroblasts show separation of the cell populations (**f**).

**Figure 4:**
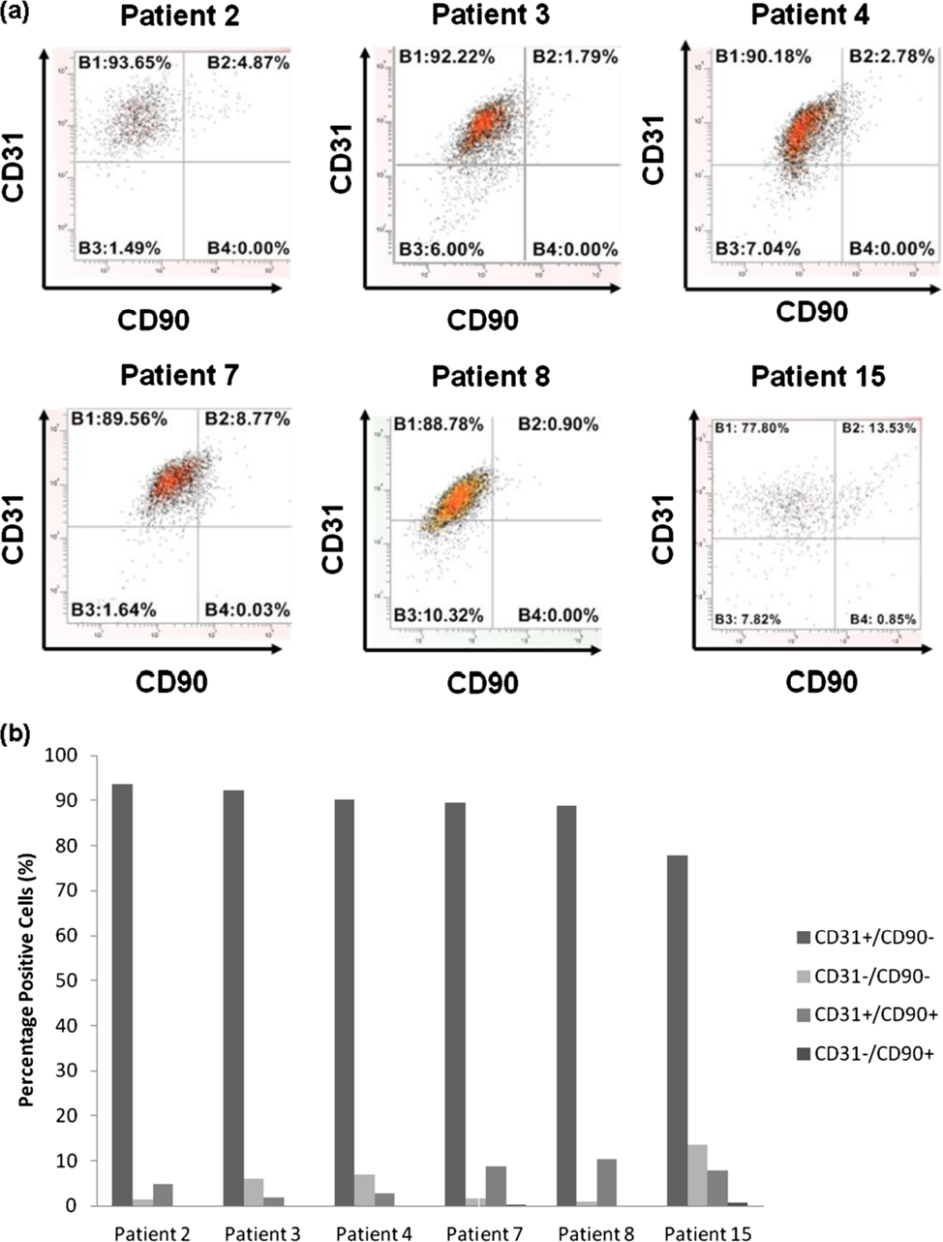
**(a) Representative flow cytometry scatter plots depicting CD31:CD90 characterisation of cells isolated from patients with emphysema and one patient free from emphysema (patient 15) via flow cytometry.** (**b**) Summary chart showing percentage of cells in the various populations.

LMVECs (Promocell) and dermal fibroblasts (gifted by ICM, Newcastle University) were used to determine the optimal concentration of each antibody (CD90 and CD31) required for flow cytometry characterisation experiments (Figure 3a-e). Once concentrations for each antibody alone were determined, a second set of experiments were conducted to determine the optimal concentration of CD31 (1 ul) and CD90 (0.5 ul) to separate a mixed population of LMVECs and fibroblasts (Figure 3f). Following these preliminary experiments, cells from patients with emphysema and a normal donor were characterised using the established protocol (Figure 4). The cell populations isolated from all donors were characterised by high expression of CD31 and low expression of CD90 (Figure 4). Cells from the normal donor (patient 15) were characterised at passage 2, as evidenced by the slightly lower number of CD31 positive cells (78%).

**Figure 5:**
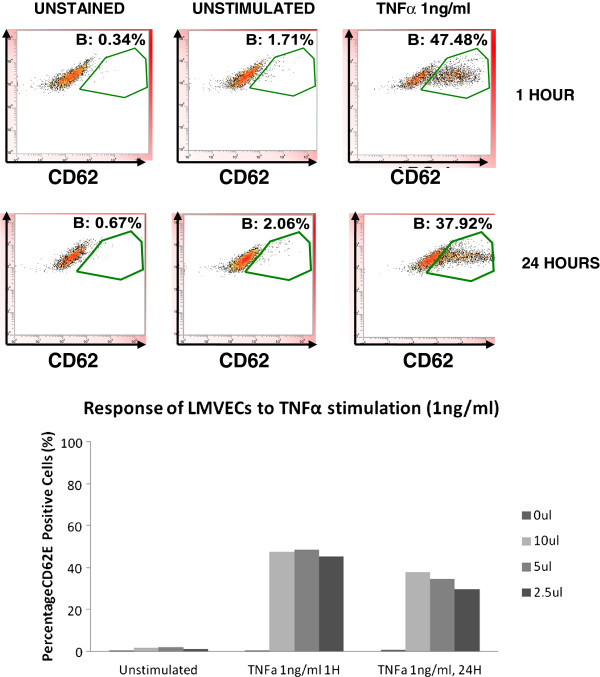
Representative flow cytometry scatter plots showing the response of LMVECs (Promocell) to TNFα (1 ng/ml) stimulation at 1 and 24 hours as detected by differing concentration of CD62E antibody (2.5-10 ul).

CD62E (E-Selectin) expression on isolated CD31 positive cells at baseline and after stimulation with TNFα was also investigated. CD62E is a cell surface adhesion molecule involved in leukocyte trafficking that is absent on microvascular endothelial cells but is inducible upon cytokine stimulation [20]. Capillaries do not express CD62E at baseline or upon activation. We therefore hypothesised that the isolated CD31 positive cells would be CD62E negative at baseline and that a proportion representing microvascular cells excluding capillaries would become CD62E positive upon stimulation while a second subpopulation representing the capillaries would remain CD62E negative. Commercially available LMVECs were first investigated to determine the concentration of TNFα, CD62 antibody and appropriate time course required. Approximately 40-50% cells stained positively for CD62E at a low concentration of TNFα (1 ng/ml) for 1 hour and 24 hours, across a range of antibody concentration (2.5 ul-10 ul) (Figure 5). A similar percentage of cells were positive for CD62E with higher concentrations of TNFα (2-8 ng/ml) (data not shown).

**Figure 6:**
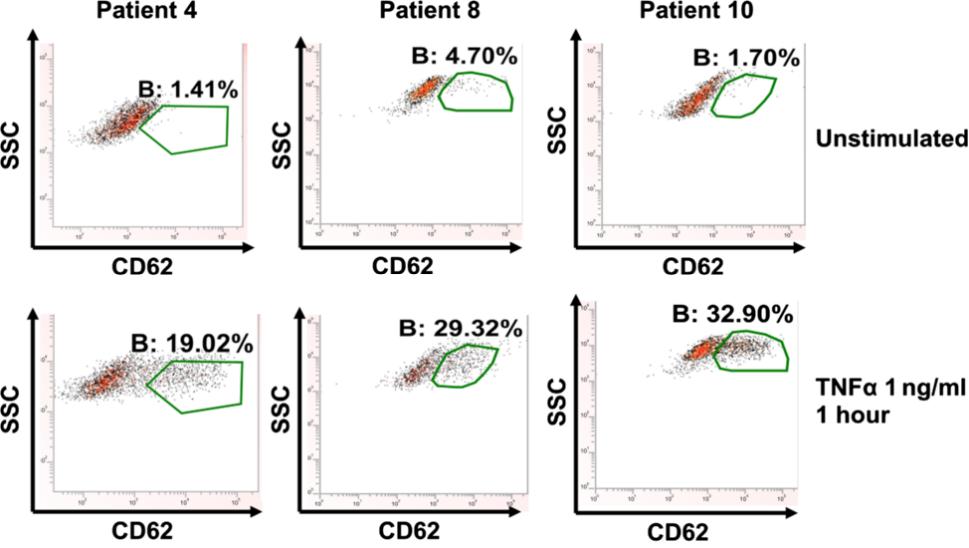
Representative scatter plots showing the response of microvascular endothelial cells from patients with emphysema to TNFα (1 ng/ml) stimulation for 1 hour as measured via CD62E immunostaining via flow cytometry.

**Figure 7:**
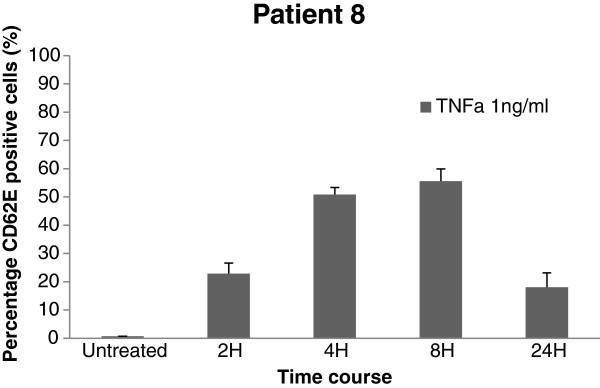
**Percentage CD62E positive cells (patient 8) determined via flow cytometry (Mean +/− SD; n = 3) following treatment with TNFα (1 ng/ml).** Untreated cells did not express CD62E. The percentage of treated cells that expressed CD62E was maximal at 8 hours before falling at 24 hours. The profile observed is most likely the result of TNFα induced transcriptional induction at early time points and cleavage of CD62E from the cell surface at 24 hours.

CD31 positive cells from 3 patients with emphysema were selected at random and thereafter stimulated with 1 ng/ml TNFα for 1 hour and stained for CD62E to investigate whether these cells were microvascular in origin. The emphysema cells demonstrated minimal baseline CD62E expression (<5%) with a rightward shift in response to TNFα stimulation at 1 hour with approximately 30% cells staining positively for CD62E (Figure 6). Cells from one emphysema donor (patient 8) were used to further investigate CD62E expression on these isolated cells at further time points (2, 4 and 8 hours) (Figure 7). There was similar induction of CD62E expression that became maximal at 8 hours and then fell at 24 hours to levels similar to previous experiments. Due to the precious nature of these cells, this time course was not repeated in multiple donors, as having demonstrated that the CD31 positive cells isolated were negative at baseline for CD62E but inducible in a proportion of cells, we had confirmed these to be of microvascular origin.

## Discussion

Microvascular endothelial cells have been isolated previously from a variety of organs including human lung, however we present the first report of a method to successfully isolate such cells from emphysematous lung tissue. This method allows *ex vivo* study of a cell population which may be key in the pathogenesis of emphysema. Importantly, the method we have developed allows the isolation and culture of large numbers of human LMVECs (Table 1) with a high success rate (71%). Furthermore, the cells could be successfully expanded, cryopreserved and later re-animated for use in future studies. Cells showed stability of phenotype to passage 7.

Certain steps were critical to the success of this method. The tissue could be stored for up to 24 hours from the time of transplant until histopathological processing, however once processing began, cell isolation had to follow immediately otherwise it was unsuccessful. Careful dissection of the large vessels and removal of pleura to prevent overgrowth by contaminating mesothelial cells was also vital. Daily observation of cell numbers and doubling time was also required in order to determine the optimal time for bead separation as time between each passage differed between donors and did not appear related to passage number or disease severity.

Endothelial cell extraction employed bead separation with magnetic dynal beads for CD31 (endothelial cell surface marker) and UEA-1 (an endothelial based lectin). Other researchers have previously reported difficulties when using CD31 dynal beads, hypothesising that disruption of cell surface CD31 by beads inhibited the cell to cell interactions required for successful growth in culture [20]. We did not encounter such problems, although doubling time immediately post bead separation was more prolonged.

By passage 4–6, cells appeared free from contaminating spindle shaped cells and were characterised according to a standard protocol developed using commercially available cells. The commercially available cells were used both to set a standard against which the cells isolated could be characterised and to ensure that precious isolated primary cells were not used for optimisation of experimental protocols. Comparing the cells isolated from patients with emphysema to those isolated from lung resection operations and commercially available cells provided a further control.

The immunocytochemical detection of cell surface markers via confocal microscopy confirmed the isolated cells were endothelial, staining positively for the endothelial marker CD31 with weak/absent staining for mesenchymal markers. This was further confirmed by flow cytometry, with cells staining positively for the endothelial cell marker CD31 and negatively for the fibroblast marker CD90. These approaches proved very cell efficient, requiring only small numbers of cells for full characterisation (~1×10^6^), thus preserving large numbers of cells for use in future studies.

Plant derived lectins have previously been employed to differentiate between microvascular and macrovascular endothelial cells [21]. We encountered difficulties with non-specific binding of the lectins *Griffonia* (*Bandeiraea*) *simplicifolia* and *Helix pomatia* previously used to differentiate between microvascular and macrovascular endothelial cells respectively both on single cells and on paraffin embedded tissue. As a result, we investigated E-selectin (CD62E) expression as an alternative method to differentiate between microvascular and macrovascular endothelial cells. E-Selectin (CD62E) and P-Selectin (CD62P) are receptor molecules for monocytes and neutrophils that are expressed on activated endothelial cells [22]. CD62E, in contrast to CD62P which is stored in Weibel-Palade bodies in endothelial cells, is transcriptionally induced on microvascular endothelial cells in response to cytokine stimulation [20]. Capillaries are not thought to express CD62E [23,24]. Thus quiescent microvascular cells do not express CD62E but following activation intraacinar arterioles and venules express CD62E, while capillaries remain negative for CD62E. In our studies, the isolated CD31 positive cells showed very low (<5%) staining for CD62E at baseline. In response to stimulation with TNFα, there was inducible staining in around 30-50% of the isolated cells at 1 hour. Inducible CD62E in response to TNFα suggests these endothelial cells are microvascular. Furthermore, the presence of a subpopulation that was endothelial (i.e. positive for CD31) but did not up-regulate CD62E in response to TNFα suggests that the cells in this subpopulation are pulmonary capillary endothelial cells. Importantly this subpopulation was greater in the cells isolated from patients with emphysema compared with commercially available cells from Promocell used in the optimisation experiments. These pulmonary microvascular endothelial cells may therefore provide a more appropriate model than the current commercially available cells.

Infection is undoubtedly the major challenge to successful isolation and investigation of human LMVECs. Due to the inherent risks of infection whilst isolating the cells, the lobe of lung was placed in media containing 1% PSA prior to processing. 1% PSA was included in all MV2 media used in cell culture, the risk of infection being deemed greater than any adverse effect on growth kinetics the antimicrobials may have. In spite of this, a number of cell cultures were lost to infection, mostly around passage 6. With cell aging, growth kinetics reduced, with greater time to confluence. Cells therefore spent a longer time in culture with each successive passage, increasing the likelihood of infection. Amphotericin was included as we encountered more fungal infections than bacterial infections.

As with all *ex vivo* cell culture systems, inherent limitations are associated. Cells were passaged 3–5 times prior to obtaining pure cobblestone cultures which were characterized as endothelial. Cells therefore have a protracted culture period, with possible associated increased senescence and change in cell characteristic. Cells were grown in MV2 media (Promocell) which included 5% fetal calf serum supplementation and other survival factors such as hydrocortisone (0.2 ug/ml), recombinant human epidermal growth factor (5 ng/ml) fibroblast growth factor (10 ng/ml) vascular endothelial growth factor (0.5 ng/ml) and insulin like growth factor (Long R3) (20 ng/ml). The addition of hydrocortisone to cell culture media has been a contentious matter due to concerns over increased cell stress and how this may change cellular physiology. The concentration of hydrocortisone in MV2 media (Promocell) is considerably lower than in other types of microvascular endothelial cell media and its omission led to cell death.

Finally, our method of cell isolation can be applied to other respiratory diseases in which the pulmonary microvasculature may be pivotal such as pulmonary arterial hypertension and idiopathic pulmonary fibrosis. Indeed we have successfully isolated large numbers of cells from these pathologies (data not shown) with higher yields of cells at lower passage than the emphysema model.

## Conclusions

In conclusion, pulmonary microvasculature endothelial cells can be isolated from severely emphysematous lungs removed at transplantation with good success (71%). Full characterisation of these cells confirms these to be of high purity and of microvascular origin. Successful culture yields large numbers of cells that can be cryopreserved and later reanimated for use in mechanistic studies. We propose that these cells provide a more biologically relevant cell model for investigating the pulmonary microvasculature and the pathogenesis of emphysema.

## Competing interest

The authors declare that they have no competing interests.

## Authors’ contributions

All authors were involved in the conception, design and planning of the study. LSM, JL, AJF and PAC obtained ethical approval and consent; Initial cell isolation technique was worked up by SD; Cell isolation was carried out by LSM, SD, IGD, WT; Characterisation experiments were performed by LSM with support from SD, IGD, WT, AJF and PAC. The manuscript was initially drafted by LSM. All authors contributed to redrafting and have read and approved the final manuscript.
